# Mental health and neurocognitive disorder–related hospitalization rates in immigrants and Canadian-born population: a linkage study

**DOI:** 10.17269/s41997-023-00740-1

**Published:** 2023-02-21

**Authors:** Anne Grundy, Edward Ng, Claudia Rank, Jacklyn Quinlan, George Giovinazzo, Rachel Viau, David Ponka, Rochelle Garner

**Affiliations:** 1 Migration Health Branch, Immigration, Refugees and Citizenship Canada, 250 Tremblay Rd, Ottawa, ON K1G 5P4 Canada; 2 Health Analysis Division, Statistics Canada, Ottawa, ON Canada; 3 Department of Family Medicine, University of Ottawa, Ottawa, ON Canada

**Keywords:** Immigrant, Mental health, Hospital discharge, Immigration admission category, Immigrant, santé mentale, hospitalisation, catégorie d’immigrant

## Abstract

**Objectives:**

Mental health and neurocognitive conditions are important causes of hospitalization among immigrants, though patterns may vary by immigrant category, world region of origin, and time since arrival in Canada. This study uses linked administrative data to explore differences in mental health hospitalization rates between immigrants and individuals born in Canada.

**Methods:**

Hospital records from the Discharge Abstract Database and the Ontario Mental Health Reporting System for 2011 to 2017 were linked to the 2016 Longitudinal Immigrant Database and to Statistics Canada’s 2011 Canadian Census Health and Environment Cohort. Age-standardized hospitalization rates for mental health–related conditions (ASHR-MHs) were derived for immigrants and the Canadian-born population. ASHR-MHs overall and for leading mental health conditions were compared between immigrants and the Canadian-born population, stratified by sex and selected immigration characteristics. Quebec hospitalization data were not available.

**Results:**

Overall, immigrants had lower ASHR-MHs compared to the Canadian-born population. Mood disorders were leading causes of mental health hospitalization for both cohorts. Psychotic, substance-related, and neurocognitive disorders were also leading causes of mental health hospitalization, although there was variation in their relative importance between subgroups. Among immigrants, ASHR-MHs were higher among refugees and lower among economic immigrants, those from East Asia, and those who arrived in Canada most recently.

**Conclusion:**

Differences in hospitalization rates among immigrants from various immigration streams and world regions, particularly for specific types of mental health conditions, highlight the importance of future research that incorporates both inpatient and outpatient mental health services to further understand these relationships.

**Supplementary Information:**

The online version contains supplementary material available at 10.17269/s41997-023-00740-1.

## Résumé

**Objectifs:**

Les problèmes de santé mentale et les troubles neurocognitifs sont des causes importantes d’hospitalisation chez les immigrants, bien que les tendances puissent varier selon la catégorie d’immigrants, la région d’origine mondiale et le temps écoulé depuis l’arrivée au Canada. Cette étude utilise des données administratives couplées afin d’explorer les différences dans les taux d’hospitalisation en santé mentale entre les immigrants et les personnes nées au Canada.

**Méthodes:**

Les dossiers hospitaliers de la Base de données sur les congés des patients et du Système d’information ontarien sur la santé mentale de 2011 à 2017 ont été couplés à la Base de données longitudinales sur l’immigration de 2016 et aux cohortes santé et environnement du Recensement canadien de 2011 de Statistique Canada. Les taux d’hospitalisation normalisés selon l’âge pour les problèmes de santé mentale (THNA-SM) ont été comparés entre les immigrants et la population née au Canada, stratifiés selon le sexe et certaines caractéristiques d’immigration. Les données sur les hospitalisations au Québec n’étaient pas disponibles.

**Résultats:**

Dans l’ensemble, les immigrants avaient des THNA-SM plus faibles que la population née au Canada. Les troubles de l’humeur étaient les principales causes d’hospitalisation en santé mentale pour les deux cohortes. Les troubles psychotiques, liés aux substances et neurocognitifs étaient également les principales causes d’hospitalisation en santé mentale, bien que leur importance relative varie entre les sous-groupes. Chez les immigrants, les THNA-SM étaient plus élevés chez les réfugiés et plus faibles chez les immigrants économiques, ceux de l’Asie de l’Est et ceux qui sont arrivés au Canada plus récemment.

**Conclusion:**

Les différences dans les taux d’hospitalisation chez les immigrants des divers groupes d’immigration et régions du monde, plus particulièrement pour certains types de problèmes de santé mentale, soulignent l’importance de recherches futures qui intègrent les services de santé mentale aux patients hospitalisés ainsi qu’aux patients externes afin de mieux comprendre ces relations.

## Introduction

Mental health is a concern at various stages of the immigration pathway, and these issues can have impacts on newcomers in terms of settlement, health, and social service needs. While some studies suggest that immigrants may use mental health services less often than non-immigrants (Abebe et al., 2017; Chen & Vargas-Bustamante, 2011; Derr, 2016; Islam et al., 2018; Ng & Zhang, 2021; Nwoke et al., 2020), there is also evidence suggesting that there may be differences based on immigrant admission category (Abebe et al., 2017; Durbin et al., 2014; Ng & Zhang, 2021) and world region of origin (Abebe et al., 2017; Durbin et al., 2015; Kirmayer et al., 2007; Ng & Zhang, 2021).

Some studies have noted differences in both the prevalence of mental health conditions (Abebe et al., 2017; Derr, 2016; Ng & Zhang, 2020; Robert & Gilkeinson, 2010) and mental health service use (Derr, 2016; Durbin et al., 2014; Ng & Zhang, 2021; Saunders et al., 2018; Vigod et al., 2017) between refugee and non-refugee immigrants and across groups of immigrants from different world regions (Abebe et al., 2017; Durbin et al., 2015; Kirmayer et al., 2007; Ng & Zhang, 2020, 2021). In a recent systematic review and meta-analysis of specific mental health conditions among refugees and asylum seekers, more than 30% had post-traumatic stress disorder or depression, suggesting that refugees may have a particular need for mental health services (Blackmore et al., 2020). Increased exposure to trauma among refugees coming from countries experiencing social turmoil has been highlighted as an important risk factor for subsequent mental health conditions (Pottie et al., 2011).

Previous analysis of hospitalization events among immigrants identified mental health conditions (including neurocognitive conditions) among the top six reasons for hospitalization (Ng et al., 2021). However, few previous Canadian studies examining the relationship between immigration and mental health (Durbin et al., 2014, 2015; Kirmayer et al., 2007; Ng & Omariba, 2010; Ng & Zhang, 2020, 2021) have examined differences by specific mental health conditions. This descriptive study expands upon previous work on all-cause hospitalizations among immigrants (Ng et al., 2021) to examine hospitalization rates for specific mental health and neurocognitive conditions among immigrants and the Canadian-born population, stratified by sex, world region of origin, and selected immigration characteristics.

## Methods

Similar to previous work (Ng et al., 2021), this study compared mental health hospitalization rates among immigrants and the Canadian-born population using linked administrative datasets at Statistics Canada (StatCan). The work was conducted using StatCan’s Social Data Linkage Environment (SDLE) and its highly secured central depository called the Derived Record Depository (DRD) (Statistics Canada, 2017).

### Data sources

Mental health hospitalization data were obtained from the Canadian Institute for Health Information’s (CIHI) Discharge Abstract Database (DAD) and the Ontario Mental Health Reporting System (OMHRS). The DAD contains demographic, administrative, and clinical data for all acute care and some psychiatric, chronic rehabilitation, and day-surgery discharges for all provinces and territories excluding Quebec (Canadian Institute for Health Information, 2018). The DAD includes hospital discharges occurring between April 1, 1994 and March 31, 2017 (comprising 84.8 million hospital discharge records) that were eligible for linkage using a deterministic approach (linkage rate of 91%; see Carrière et al. (2018) for details). The OMHRS is a CIHI database that includes information about individuals admitted to general and speciality facilities in Ontario since April 2006 (Canadian Institute for Health Information, 2016b). This supplemented the absence of mental health hospitalization data from the DAD from Ontario since 2006/2007. The OMHRS includes information at the assessment level about patients’ mental and physical health, social supports, and service use. OMHRS records occurring between April 1, 2006 and March 31, 2018 were eligible for linkage in the present study (*n*=1,248,844), using a deterministic approach (linkage rate of 82.7%; see Social Data Linkage Environment, Analysis and Data Development Section, Special Surveys Division (2019) for details).

Immigrant records were obtained from the Longitudinal Immigration Database (IMDB), which is derived from the Immigration Landing files provided to StatCan by Immigration, Refugees and Citizenship Canada (IRCC). The 2016 IMDB contains administrative information since 1980 for all individuals who landed as permanent residents or had temporary resident permits issued. In the present study, landing records and temporary resident permits between 1980 and 2017 were eligible for linkage (*n*=12,317,708). Using probabilistic methods, the 2016 IMDB was linked to the DRD (linkage rate of 90%, *n*=11,036,264; see Social Data Linkage Environment, Analysis, and Data Development Section, Special Surveys Division (2019) for details).

Data on Canadian-born individuals were obtained from the 2011 Canadian Census Health and Environment Cohort (CanCHEC), a population-based study cohort based on the 2011 Canadian National Household Survey (NHS) that was probabilistically linked to administrative health data (e.g., mortality, hospitalizations, and cancer diagnoses) (Health Analysis Division, 2019). The NHS was probabilistically linked to the DRD with a linkage rate of 96.7% (*n*=6,496,380; see Health Analysis Division (2019) for details).

Together, these linkages provide the corresponding hospitalization outcomes among the Canadian-born cohort based on the 2011 NHS-DAD-OMHRS linked database, while the IMDB was linked to hospitalization data for the immigrant cohort, based on the 2016 IMDB-DAD-OMHRS linked database.

### Study cohorts

**Fig. 1:**
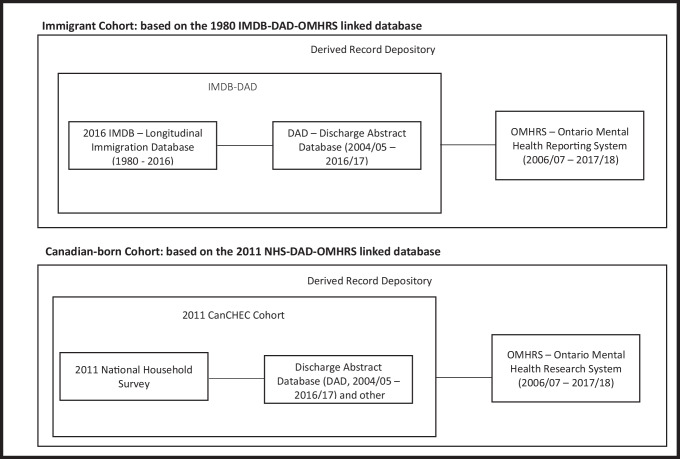
Data sources and linkages for immigrant and Canadian-born cohorts

Similar to previous work on all-cause hospitalization, this study included two cohorts—an immigrant cohort and a Canadian-born cohort (Fig. 1)—details of which have been previously described (Ng et al., 2021). Briefly, the immigrant cohort was limited to permanent residents who arrived in Canada between January 1, 1980, and May 10, 2011: temporary residents (e.g., students, workers) were excluded. To account for the fact that Quebec does not contribute data to the DAD, immigrants who landed in Quebec were excluded from the analysis. The final number of individuals in the immigrant cohort was 4,162,005.

The Canadian-born cohort was based on respondents to the 2011 CanCHEC who self-identified as being Canadian-born. Similar to the approach taken for the immigrant cohort, 2011 CanCHEC members who resided in Quebec were excluded from the analysis. The final sample size of the Canadian-born cohort was 3,754,230 representing a weighted population of 19,037,385 individuals.

Acute care hospitalization records from the DAD and OMHRS were linked to the immigrant and Canadian-born cohorts for a 5-year follow-up, starting from Census Day 2011 (May 10, 2011) through May 9, 2016 (Supplementary Fig. 1).

### Hospital discharges for mental health–related conditions

The primary study outcome was a hospital discharge related to a mental health or neurocognitive condition during the follow-up period. DAD-derived acute care hospital discharges were classified according to the condition most responsible for the patient’s hospitalization (most responsible diagnosis; MRDX) (Canadian Institute for Health Information, 2016a). The MRDX was chosen to capture cause of hospitalization, as other potential indicators such as medication use were not available in the DAD. The MRDX in DAD is coded using the 10th edition of the *International Classification of Diseases and Related Health Problems, Canada* (ICD-10-CA) and these were first subdivided according to their chapter code, with a special focus on codes F01 to F99 from chapter 5 for Mental, Behavioral, and Neurodevelopmental disorders (Supplementary Table 1). Instead of ICD-10-CA coding, diagnoses are coded in the OMHRS using versions IV and V of the *Diagnostic and Statistical Manual of Mental Disorders* (DSM). To maintain consistency in diagnostic classifications between DAD and OMHRS, a CIHI-developed classification crosswalk (Canadian Institute for Health Information, 2021) was used to categorize the mental health–related hospitalizations from the two data sources into the following groups: (1) substance-related and addictive disorders; (2) psychotic disorders (includes schizophrenia spectrum and other psychotic disorders); (3) mood disorders (which includes bipolar and related disorders, depressive disorders, and other mood disorders); (4) anxiety disorders; (5) personality disorders; (6) neurocognitive disorders; and (7) other mental health conditions (Canadian Institute for Health Information, 2021). In addition, DAD records that were coded as self-inflicted injuries and poisonings were classified as self-harm (X60–X84 in ICD10-CA). Noting that CIHI includes neurocognitive conditions as a subgroup of mental health conditions, we will use the term “mental health” to include neurocognitive conditions in this paper.

To avoid over-estimation of discharges due to hospital transfers, hospital admissions that occurred within 1 day of a previous discharge for the same patient were consolidated into a single hospitalization episode: in these cases, the MRDX for the last hospital discharge within the episode was used to characterize the nature of the hospitalization.

### Covariates

Age and sex were determined for the immigrant and Canadian-born cohorts based on IMDB and CanCHEC, respectively. For the Canadian-born cohort, age was calculated as of May 10, 2011. Among immigrants, age was calculated as the difference between 2011 and the birth year obtained from IMDB. Age was subsequently grouped for both cohorts as 0–17, 18–39, 40–64, and 65 years and older.

Additional characteristics were included for the 2016 IMDB-DAD-OMHRS immigrant cohort. To reflect time since immigration to Canada, landing year was grouped as 1980–1989, 1990–2002, and 2003–2011. These cut-points were chosen to allow immigrants in the final group to be those who arrived under the most recent version of the *Immigration and Refugee Protection Act*, which was passed in 2001 (Immigration and Refugee Protection Act, 2001). Immigrants’ birth country was grouped into 10 world regions: the United States; Caribbean, Central, and South America; Western Europe; Eastern Europe; Sub-Saharan Africa; Southwest Asia and North Africa; South Asia; Southeast Asia; East Asia; and Others (Ng, 2011). As previously described (Ng et al., 2016), immigrant admission categories were classified into four general groups in accordance with the Immigration and Refugee Protection Act (2001): economic class; family class; refugees; and others.

### Statistical methods

Descriptive statistics (frequency tables and proportions) were used to describe the immigrant and Canadian-born cohorts. Crude and age-standardized mental health–related hospitalization rates (ASHR-MHs) were derived by sex for mental health–related hospitalizations overall, and for the three leading causes (as determined by distribution) among the immigrant cohort. These rates were also examined within the immigrant cohort by the variables identified above (i.e., immigrant admission category, landing year, and world region of birth). The Canadian-born population (excluding Quebec) for the 2011 NHS was used as the reference population for age standardization. Rate derivation was adjusted for individuals who died during follow-up. Last, to account for the 2011 NHS’s complex survey design (Statistics Canada, 2011), and to adjust for linkage, measures for the Canadian-born cohort were calculated using the CanCHEC’s sample and bootstrap weights (Health Analysis Division, 2019). No weighting adjustment was used for the immigrant cohort, as the cohort is considered to be a 100% census of immigrants, such that no sampling adjustment is required.

## Results

Among immigrants, the highest percentages were admitted under the economic class (51%) followed by the family class (33%). Almost half (45%) of these immigrants were aged 40–64 years in 2011, compared to 34% for the Canadian-born population. Among immigrants, almost half (46%) arrived in Canada between 1990 and 2002 and more than half (53%) were from South, Southeast, or East Asia (Table 1).

**Table 1:** Descriptive characteristics of the study cohort

	Canadian-born cohort		Immigrant cohort	
	Males (*n* = 9,456,690)	Females (*n* = 9,580,695)	Males (*n* = 1,990,725)	Females (*n* = 2,171,280)
Age groups				
0–17	26.6%	24.7%	10.7%	9.3%
18–39	29.5%	28.7%	35.4%	37.1%
40–64	33.2%	34.0%	46.2%	44.5%
65+	10.8%	12.6%	7.6%	9.1%
Immigration landing year				
1980–1989			16.9%	16.4%
1990–2002			45.6%	45.9%
2003–2011			37.5%	37.6%
Source world region				
USA			2.6%	2.9%
Caribbean/Central and South America			9.8%	10.5%
Western Europe			7.5%	6.6%
Eastern Europe			10.6%	10.8%
Sub-Saharan Africa			5.5%	5.1%
Southwest Asia with North Africa			10.9%	9.1%
South Asia			23.2%	21.8%
Southeast Asia			8.9%	11.0%
East Asia			19.9%	21.3%
Other			1.2%	1.1%
Immigrant admission categories				
Economic			53.9%	48.8%
Family			28.2%	37.5%
Refugees			16.7%	12.5%
Others^a^			1.3%	1.3%

^a^Foreign nationals admitted on humanitarian and compassionate grounds who do not qualify in any immigration category

Subtotals may not add up to total due to miscellaneous rounding

Source: The 2011 NHS-DAD-OMHRS linked data and the 2016 IMDB-DAD-OMHRS linked database

### Leading causes of mental health hospitalization

Among immigrant and Canadian-born males, the most prevalent categories of mental health–related hospitalization were psychotic disorders, substance-related and addictive disorders, and mood disorders (Table 2). However, the proportions and relative order of these three categories differed between the two groups. The leading cause of mental health–related hospitalization among immigrant males was psychotic disorders (34%), while for Canadian-born males it was mood disorders (25%). Overall, the top three causes of mental health–related hospitalization made up a greater share of all mental health–related hospitalizations among immigrant (78%) compared to Canadian-born males (65%).

**Table 2:** Percentage and ranking distribution of mental health–related hospitalization events during 5-year follow-up among the Canadian-born and immigrant cohorts outside of Quebec

Mental health conditions	Canadian-born cohort				Immigrant cohort			
	Males (*n*^a^ = 222,265)		Females (*n*^a^ = 247,960)		Males (*n*^a^ = 29,285)		Females (*n*^a^ = 26,740)	
	*%*	*Rank*	*%*	*Rank*	*%*	*Rank*	*%*	*Rank*
Substance use and addictive disorders	22.3	2	11.9	2	24.2	2	6.6	4
Psychotic disorders	18.5	3	10.1	4	33.6	1	27.9	2
Mood disorders	24.6	1	34.5	1	20.0	3	34.1	1
Anxiety disorders	3.4	5	5.4	6	1.7	6	3.3	6
Personality disorders	1.5	7	4.0	7	0.9	7	2.0	7
Neurocognitive disorders	10.8	4	11.5	3	7.3	4	9.4	3
All others in ICD Ch. 5^b^	15.7	n/a	16.9	n/a	10.0	n/a	12.7	n/a
Self-harm from ICD Ch. 10	3.1	6	5.6	5	2.5	5	4.1	5

^a^Refers to number of hospitalization events

^b^This category was not ranked due to heterogeneous mix of conditions

Source: The 2011 NHS-DAD-OMHRS linked data and the 2016 IMDB-DAD-OMHRS linked database

Among immigrant and Canadian-born females, mood disorders were the leading cause of mental health–related hospitalization, accounting for approximately 34% of mental health–related hospitalizations (Table 2). Psychotic disorders (25%) were the second leading cause among immigrant females, while substance-related and addictive disorders (12%) were the second leading cause for Canadian-born females. In both groups, neurocognitive disorders were the third leading cause of mental health–related hospitalization. The top three causes comprised 71% of all mental health–related hospitalizations among immigrant females but only 57% of mental health–related hospitalizations among Canadian-born females (Table 2).

### Comparison of overall ASHR-MHs

The overall crude and ASHR-MHs, stratified by sex, are shown in Table 3. Among both males and females, the ASHR-MH among immigrants was almost half that of the Canadian-born cohort. The ASHR-MHs for immigrants were lowest in the economic class, followed by the family and refugee classes. In both males and females, ASHR-MH was lowest for those who landed in more recent years and also for those from East Asia.

**Table 3:** Overall crude and age-standardized acute care mental health–related hospitalization rate^a^ (ASHR-MH, per 10,000 population) by sex and by selected immigrant characteristics, Canadian-born and immigrant cohorts outside of Quebec

	Males			Females		
	Crude rate	ASHR-MH	95% confidence interval	Crude rate	ASHR-MH	95% confidence interval
NHS Canadian-born population (weighted)	47.7	48.2	46.7–49.7	52.4	52.3	51.0–53.7
1980–2011 IMDB immigrants	29.6	28.4	27.8–29.0	24.8	25.6	25.0–26.2
Immigrant admission category						
Economic	18.7	18.7	18.0–19.3	18.4	19.6	18.9–20.4
Family	40.5	38.4	36.6–40.2	28.4	30.8	29.3–32.4
Refugee	46.2	43.9	41.8–46.0	37.6	39.0	36.9–41.1
Other^b^	34.8	34.2	27.4–41.1	39.1	38.7	30.5–46.9
Immigration landing year						
1980–1989	37.8	30.0	28.3–31.6	34.5	27.4	26.0–28.9
1990–2002	34.8	34.3	33.0–35.5	26.8	29.7	28.5–30.9
2003–2011	19.7	18.7	17.9–19.4	18.1	18.2	17.5–19.0
Source world region						
USA	30.1	32.2	28.4–36.0	36.3	36.4	32.3–40.4
Caribbean/Central and South America	40.6	41.2	38.3–44.1	34.1	39.2	36.5–41.9
Western Europe	30.5	30.3	27.9–32.7	37.7	38.0	35.2–40.7
Eastern Europe	41.0	39.2	36.2–42.2	34.5	36.6	33.8–39.5
Sub-Saharan Africa	46.2	43.8	40.4–47.1	40.2	39.6	36.2–43.0
Southwest Asia with North Africa	33.9	31.9	30.0–33.9	25.7	25.2	23.5–26.9
South Asia	32.6	29.4	28.1–30.7	19.9	19.9	18.9–20.9
Southeast Asia	18.2	18.8	17.3–20.3	15.3	16.9	15.4–18.3
East Asia	12.1	13.3	12.3–14.4	15.3	16.4	15.4–17.4

^a^Standardization used the overall 2011 NHS population outside of Quebec as the reference population

^b^Foreign nationals admitted on humanitarian and compassionate grounds who do not qualify in any immigration category

Source: The 2011 NHS-DAD-OMHRS linked data and the 2016 IMDB-DAD-OMHRS linked database

### Cause-specific ASHR-MHs

Using the three leading causes of mental health–related hospitalizations among immigrant males, the crude rates and ASHR-MHs for the top three mental health–related conditions for Canadian-born and immigrant males are shown in Table 4. For both substance-related disorders and mood disorders, immigrant males had lower ASHR-MHs than Canadian-born males. However, for psychotic disorders, immigrant males had a comparable rate to Canadian-born males per 10,000 population (8.9, 95% CI=8.5–8.9 vs 8.7, 95% CI=8.5–9.4, respectively).

**Table 4:** Leading cause-specific crude and age-standardized hospitalization rate^a^ (ASHR, per 10,000 population) by selected characteristics, Canadian-born and immigrant males outside of Quebec

	Psychotic disorders (rank #1)			Substance-related and addictive disorders (rank #2)			Mood disorders (rank #3)		
	Crude rate	ASHR	95% confidence interval	Crude rate	ASHR	95% confidence interval	Crude rate	ASHR	95% confidence interval
NHS Canadian-born population (weighted)	8.8	8.7	8.0–9.4	10.6	10.7	10.1–11.2	11.7	11.7	11.2–12.2
1980–2011 IMDB immigrants	9.9	8.9	8.5–9.2	7.2	6.0	5.7–6.2	5.9	5.6	5.4–5.8
Immigrant admission category									
Economic	6.0	5.5	5.1–5.8	3.4	3.0	2.8–3.3	4.8	4.6	4.4–4.9
Family	11.8	10.8	9.8–11.7	11.9	10.1	9.3–10.8	7.0	7.0	6.5–7.6
Refugee	19.3	17.6	16.2–19.0	11.2	9.2	8.4–9.9	8.0	7.4	6.7–8.1
Other^b^	10.6	11.4	7.2–15.5	5.9	6.0	3.9–8.1	4.1	4.0	2.4–5.6
Immigration landing year									
1980–1989	10.4	9.3	8.2–10.5	10.4	7.5	6.8–8.2	7.3	5.3	4.8–5.7
1990–2002	12.4	11.7	11.0–12.4	8.7	7.3	6.8–7.7	6.7	6.9	6.5–7.4
2003–2011	6.7	5.5	5.1–5.9	3.8	3.3	3.0–3.6	4.3	4.2	3.9–4.5
Source world region									
USA	5.7	5.8	4.2–7.5	6.0	6.2	4.9–7.6	8.9	9.1	7.2–11.0
Caribbean/Central and South America	17.7	15.7	13.7–17.7	7.9	6.8	5.9–7.7	7.1	7.3	6.4–8.3
Western Europe	5.4	5.5	4.4–6.5	6.2	5.1	4.4–5.9	9.1	8.1	7.1–9.0
Eastern Europe	12.4	11.3	9.9–12.7	12.5	10.3	9.2–11.4	8.3	7.8	6.9–8.7
Sub-Saharan Africa	24.5	20.7	18.5–22.9	6.5	5.6	4.7–6.4	7.9	7.7	6.4–9.0
Southwest Asia with North Africa	13.5	11.6	10.4–12.8	4.9	4.2	3.6–4.9	7.8	7.2	6.4–8.0
South Asia	8.5	7.3	6.7–7.9	12.9	10.4	9.6–11.2	5.2	4.7	4.3–5.1
Southeast Asia	6.8	5.9	5.1–6.8	3.2	3.0	2.4–3.5	3.6	3.6	3.0–4.2
East Asia	4.3	4.0	3.4–4.5	0.7	0.7	0.5–0.9	2.8	3.2	2.8–3.7

^a^Standardization used the overall 2011 NHS population outside of Quebec as the reference population

^b^Foreign nationals admitted on humanitarian and compassionate grounds who do not qualify in any immigration category

Source: The 2011 NHS-DAD-OMHRS linked data and the 2016 IMDB-DAD-OMHRS linked database

Among immigrant males, economic immigrants had the lowest ASHR-MH for all three leading causes compared to family class immigrants and refugees. Refugees had higher ASHR-MHs, particularly for psychotic disorders. By world region of origin, those from the Caribbean and Sub-Saharan Africa had elevated ASHR-MH for psychotic disorders, but relatively low ASHR-MH for substance-related disorders, compared to immigrant males from other world regions.

Table 5 shows the crude and ASHR-MH for the three leading causes of mental health–related hospitalizations among Canadian-born and immigrant females. For both mood and neurocognitive disorders, immigrant females had lower ASHR-MHs compared to Canadian-born females. However, similar to the pattern seen among males, the ASHR-MH for psychotic disorders was similar between immigrant and Canadian-born females (5.8, 95% CI=5.8–6.1 vs 5.3, 95% CI=4.9–5.6 per 10,000 population, respectively). Among female immigrants, those admitted under the economic class also had the lowest ASHR-MH for all three leading causes of mental health–related hospitalization compared to those admitted under the family and refugee classes. In particular, refugees had high ASHR-MHs for mood disorders and psychotic disorders. Immigrant females from the Caribbean and from sub-Saharan Africa had elevated ASHR-MH for psychotic disorders compared to female immigrants from other world regions. For mood disorders, the ASHR-MHs were comparable across multiple regions, although those from the USA and from Western Europe had ASHR-MHs most comparable to those of Canadian-born females.

**Table 5:** Leading cause-specific crude and age-standardized hospitalization rate^a^ (ASHR, per 10,000 population) by selected characteristics, Canadian-born and immigrant females outside of Quebec

	Mood disorders (rank #1)			Psychotic disorders (rank #2)			Neurocognitive disorders (rank #3)		
	Crude rate	ASHR	95% confidence interval	Crude rate	ASHR	95% confidence interval	Crude rate	ASHR	95% confidence interval
NHS Canadian-born population (weighted)	18.1	18.1	17.5–18.7	5.3	5.3	4.9–5.6	6.0	5.9	5.6–6.2
1980–2011 IMDB immigrants	8.4	8.5	8.2–8.8	6.9	5.8	5.6–6.1	2.3	2.9	2.8–3.1
Immigrant admission category									
Economic	6.8	7.0	6.6–7.4	5.1	4.5	4.2–4.8	0.6	1.6	1.4–1.8
Family	9.4	10.7	9.8–11.5	7.3	6.1	5.7–6.6	4.5	3.2	3.1–3.4
Refugee	12.0	12.1	11.1–13.1	12.5	10.4	9.5–11.4	1.8	3.1	2.6–3.6
Other^b^	10.3	11.6	7.7–15.5	9.0	8.3	5.4–11.2	10.0	4.3	3.4–5.1
Immigration landing year									
1980–1989	10.9	8.8	8.1–9.6	9.2	7.7	6.9–8.5	6.1	4.1	3.8–4.4
1990–2002	9.1	10.3	9.7–10.9	8.2	6.9	6.5–7.3	2.4	2.8	2.6–2.9
2003–2011	6.6	6.4	6.0–6.7	4.3	3.7	3.4–4.0	0.6	1.5	1.3–1.7
Source world region									
USA	15.2	14.7	12.5–16.8	4.5	4.2	3.2–5.3	1.9	3.3	2.4–4.3
Caribbean/Central and South America	11.3	13.4	11.9–15.0	10.8	8.8	7.9–9.8	2.9	3.7	3.2–4.2
Western Europe	15.2	14.4	13.0–15.9	4.3	4.2	3.2–5.1	4.2	4.9	4.2–5.5
Eastern Europe	11.4	11.2	10.1–12.4	9.2	7.3	6.5–8.1	3.3	4.1	3.6–4.6
Sub-Saharan Africa	12.2	12.4	10.8–14.1	15.8	12.4	10.9–14.0	2.0	3.9	3.0–4.7
Southwest Asia with North Africa	8.6	8.1	7.3–8.9	6.8	5.8	5.0–6.6	1.4	2.4	1.9–2.8
South Asia	7.0	6.8	6.2–7.3	5.9	5.0	4.6–5.5	2.2	2.6	2.4–2.9
Southeast Asia	4.9	5.4	4.7–6.1	4.8	4.1	3.5–4.6	1.4	2.0	1.7–2.3
East Asia	4.8	5.3	4.8–5.8	5.1	4.4	4.0–4.9	2.0	2.1	1.9–2.3

^a^Standardization used the overall 2011 NHS population outside of Quebec as the reference population

^b^Foreign nationals admitted on humanitarian and compassionate grounds who do not qualify in any immigration category

Source: The 2011 NHS-DAD-OMHRS linked data and the 2016 IMDB-DAD-OMHRS linked database

## Discussion

This study compared mental health hospitalization rates overall and for specific mental health conditions for immigrants and Canadian-born individuals, stratified by sex, world region of origin, and several immigration characteristics. Overall, the ASHR-MHs were lower among immigrants than among the Canadian-born cohort. Among immigrants, ASHR-MHs were higher for refugees and lower for economic immigrants, those who landed more recently, and those from East Asia.

The observed lower ASHR-MH among immigrants, together with increasing ASHR-MHs the longer immigrants have spent living in Canada, is consistent with a healthy immigrant effect for mental health observed in previous studies (Abebe et al., 2017; Chen & Vargas-Bustamante, 2011; Derr, 2016; Elamoshy & Feng, 2018; Islam et al., 2018; Ng & Omariba, 2010; Ng & Zhang, 2020; Nwoke et al., 2020; Salami et al., 2017; Wu & Schimmele, 2005). However, unlike many studies that include in- and outpatient mental health service use (Abebe et al., 2017; Chen & Vargas-Bustamante, 2011; Derr, 2016; Durbin et al., 2014, 2015; Islam et al., 2018; Kirmayer et al., 2007; Nwoke et al., 2020), our study exclusively examined mental health–related hospital discharges, and therefore, may have greater representation of individuals with severe mental health conditions. Previous studies have hypothesized that some of the observed healthy immigrant effect for mental health could be due to a lack of access to mental health care services or stigma related to seeking care (Chen & Vargas-Bustamante, 2011; Derr, 2016; Durbin et al., 2014, 2015; Islam et al., 2018; Nwoke et al., 2020). As the majority of mental health care in Canada is delivered in an outpatient setting, severe events could more likely result in hospitalization. In these instances, the serious impact of mental illness on the individual’s daily life could outweigh their reluctance to seek care due to stigma. More research is needed to determine whether the lower rates of hospitalization among immigrants reflect lower rates of mental illness, decreased awareness or recognition of symptoms of mental illness (Javed et al., 2021), or unmet mental health needs.

Among immigrants, those admitted under the economic class had the lowest ASHR-MH, while rates among family and refugee class immigrants were substantially higher and closer to those observed among Canadian-born individuals. Previous studies of mental health service use in Canada have shown higher utilization rates for refugees than for non-refugees (Derr, 2016; Durbin et al., 2014; Saunders et al., 2018; Vigod et al., 2017). Also, elevated rates of mental illness among refugees and asylum seekers have been shown to persist for several years after arrival (Blackmore et al., 2020). Exposures to multiple stressors or traumatic events have been hypothesized as a potential explanation for these findings (Blackmore et al., 2020) as experiences prior to and during the refugee migration process may contribute to the higher observed ASHR-MH.

Psychotic disorders were the leading and second highest cause of mental health–related hospitalization for male (34%) and female immigrants (28%), respectively. However, psychotic disorders were not among the top three causes of mental health–related hospitalizations among Canadian-born females (accounting for only 10% of mental health–related hospitalizations) and accounted for only 18% of mental health–related hospitalizations among Canadian-born men. For both males and females, the overall ASHR-MHs for psychotic disorders were similar for immigrants and Canadian-born individuals. This pattern was mainly driven by high ASHR-MHs among refugees, as ASHR-MHs among economic and family class immigrants were either similar to or lower than those observed among the Canadian-born cohort. Most previous studies of mental health service use in Canada have not described the specific conditions being treated (Durbin et al., 2014, 2015; Islam et al., 2018; Kirmayer et al., 2007; Nwoke et al., 2020), such that direct comparisons of utilization rates related to psychotic disorders are not possible. However, the current results are consistent with a recent analysis of psychotic disorders among immigrants and refugees in Ontario, which found that the overall incidence of psychotic disorders was similar in the immigrant and general Canadian populations, while elevated rates were seen among refugees (Anderson et al., 2015). Increased rates of psychotic disorders among refugees could be a result of adverse and stressful experiences in childhood and adulthood (Beards et al., 2013; Matheson et al., 2013), and also due to difficulties in diagnosing mental health conditions in this population. For example, post-traumatic stress disorder and adjustment disorder can be particularly challenging to identify and these conditions can be misdiagnosed as psychotic disorders (Adeponle et al., 2012; Cloitre et al., 2014), which could contribute to elevated rates among refugees.

Neurocognitive disorders were among the top causes of mental health hospitalizations among immigrant and Canadian-born females, but not among males. Mental health conditions in this category include diagnoses such as dementia. This study’s finding is consistent with a higher prevalence of diseases such as dementia in women. Specifically, a report from the Alzheimer’s Society of Canada indicated that, in 2014, women represented 65% of dementia cases among those over the age of 65 (Chambers et al., 2016).

Similar to results from a previous study of all-cause hospitalization in this population (Ng et al., 2021), the ASHR-MHs were lowest among immigrants from East Asia. As in the previous study (Ng et al., 2021), this observation may be partially attributable to the fact that a substantial proportion of immigrants from East Asia in the cohort were economic class applicants, among whom the ASHR-MHs were lowest. In addition, previous studies have suggested that there may be higher levels of stigma related to mental health conditions among immigrants from East Asia that may include a failure to recognize a mental health issue (Abe-Kim et al., 2007; Chen et al., 2009; Chen & Kazanjian, 2005; Durbin et al., 2015; Javed et al., 2021; Lee et al., 2011; Tiwari & Wang, 2006). If seeking care is associated with subsequent hospitalization, a failure to recognize a mental health issue or a reluctance to seek care could be consistent with lower hospitalization rates. Previous studies have shown lower rates of mental health service use among immigrants from East Asia (Abe-Kim et al., 2007; Chen et al., 2009; Chen & Kazanjian, 2005; Derr, 2016). However, if more serious conditions are more likely to disrupt daily life and lead to care-seeking resulting in hospitalization, the potential impacts of stigma may have been minimal.

The proportion of mental health hospitalizations attributed to substance use and addictive disorders was higher among males than among females for both the Canadian-born (22.3% vs 11.9%) and immigrant (24.2% vs 6.6%) cohorts. The observation of a higher prevalence of these disorders in males than in females is consistent with other data from both Canada (Pearson et al., 2013) and other countries (Grant et al., 2004; Seedat et al., 2009). These differences may be the result of differential access to substances, where a recent review indicated that men are generally more likely than women to have access to substances (McHugh et al., 2018). In addition, although some studies have indicated that women may present to treatment more quickly after disorder onset, they also perceive more barriers to treatment than men and overall treatment rates are slightly lower among women (McHugh et al., 2018).

### Strengths and limitations

This study is the first to use the IMDB-DAD-OMHRS linked database to examine the distribution of cause-specific mental health–related hospitalizations for immigrants across Canada. The inclusion of data from almost all regions allowed for a large-scale population-based assessment of mental health in immigrants and greater overall scope compared to studies that have focused on smaller jurisdictions or specific settings (Chen et al., 2008, 2009; Durbin et al., 2014, 2015; Islam et al., 2018; Kirmayer et al., 2007). Detailed immigration data from the IMDB also allowed for assessment of mental health hospitalization by unique migration-related variables.

Despite these strengths, some limitations are to be noted. For one, the data are not fully nationally representative due to the exclusion of data from Quebec. In addition, this study did not consider potential barriers to accessing mental health services among immigrant or Canadian-born populations. As such, it is not possible to assess whether higher rates of hospitalization among the Canadian-born cohort represent true differences in the prevalence of specific mental health conditions between these populations, or whether they reflect varying access to services that might lead to subsequent hospitalization. Previous studies have found barriers to mental health care among immigrant populations, including knowledge of and access to existing services, failure to recognize mental health issues, and potential social stigma associated with using services (Chen et al., 2008, 2009; Chen & Vargas-Bustamante, 2011; Derr, 2016; Durbin et al., 2014, 2015; Islam et al., 2018; Javed et al., 2021; Nwoke et al., 2020; Saunders et al., 2018). However, most of this work has focused on primary care and specialist services delivered in the community and it is not clear whether the same barriers would apply to hospitalization.

Additional confounders could further explain differences between the immigrant and Canadian-born cohorts, and among immigrants themselves. As described earlier, the number of applications to different immigration streams varies by country of origin, such that it is not clear to what extent immigrant category or region of origin may have the most influence on observed trends. Similar confounding could also apply across other immigration-related characteristics.

Finally, it is possible that some immigrants who arrived in Canada in previous years were no longer living in the country at the beginning of the follow-up period due to emigration or death. The net effect of including such individuals in the analysis would be an underestimation of the ASHR-MH among immigrants. As described in earlier work (Ng et al., 2021), death certificates were used to adjust for mortality and tax filing data accounted for emigration in the immigrant cohort (Bérard-Chagnon, 2018; Ng et al., 2021), minimizing potential underestimation of hospitalization rates among immigrants.

## Conclusion

This study demonstrated that the rate of mental health hospitalizations was lower among immigrants than among the Canadian-born population but that the highest rates of mental health hospitalization were among immigrants in the refugee and, to a lesser extent, family class. These findings could be supplemented with data on mental health service use at various access points along the care pathway to identify vulnerable groups who may benefit from additional mental health supports, to improve immigration settlement outcomes, and to potentially reduce mental health hospitalization rates.

## Contributions to knowledge

What does this study add to existing knowledge?

- This study highlights that age-standardized mental health–related hospitalization rates are lower among immigrants than among Canadian-born individuals.
- Within the immigrant groups, rates are highest in refugees.
- Among immigrants, psychotic and mood disorders were found to be important causes of mental health–related hospitalizations.

What are the key implications for public health interventions, practice, or policy?

- Higher mental health–related hospitalization rates among refugees suggest that this is a group that could benefit from additional mental health supports to improve settlement outcomes.

## Supplementary Information

ESM 1 (DOCX 35 kb)

## Data Availability

Earlier versions of the Longitudinal Immigration Database (IMDB) linked to Hospital Discharge Abstract Database (DAD) are available through Statistics Canada’s Research Data Centres. The other data sources, such as the linkage of the IMDB to the Ontario Mental Health Reporting System, are available at the headquarters of Statistics Canada.

## Code availability

Available upon request.
