# Supplementary material for: Mental health and neurocognitive disorder–related hospitalization rates in immigrants and Canadian-born population: a linkage study
Source: Can J Public Health. 2023 Feb 21;114(4):692–704. doi: 10.17269/s41997-023-00740-1 (PMC10348999; doi:10.17269/s41997-023-00740-1)
Supplement: Supplementary file 1 — (DOCX 35 kb) [file 41997_2023_740_MOESM1_ESM.docx]

Hospital records linked to Immigrant and Canadian-born cohort to calculate hospitalization rates

Landing dates for immigrants included in Immigrant cohort

May 9, 2016

January 1, 1980

CENSUS DAY 2011^a^

May 10, 2011

^a^Members of the Canadian-born cohort were identified from the CanCHEC cohort, a population based on the 2011 National Household Survey conducted in conjunction with the 2011 Canadian Census, with reference date of May 10, 2011 (2011 Census date).

**Supplementary Figure 1:** Timelines for data linkage and follow-up.
